# Forest structure drives changes in light heterogeneity during tropical secondary forest succession

**DOI:** 10.1111/1365-2745.13680

**Published:** 2021-05-27

**Authors:** Tomonari Matsuo, Miguel Martínez‐Ramos, Frans Bongers, Masha T. van der Sande, Lourens Poorter

**Affiliations:** ^1^ Forest Ecology and Forest Management Group Wageningen University Wageningen The Netherlands; ^2^ Instituto de Investigaciones en Ecosistemas y Sustentabilidad Universidad Nacional Autónoma de México Morelia Michoacán México

**Keywords:** determinants of plant community diversity and structure, forest structure, horizontal light heterogeneity, light attenuation rate, secondary succession, tropical forest, understorey light availability, vertical light profile

## Abstract

1. Light is a key resource for tree performance and hence, tree species partition spatial and temporal gradients in light availability. Although light distribution drives tree performance and species replacement during secondary forest succession, we yet lack understanding how light distribution changes with tropical forest development.
2. This study aims to evaluate how changes in forest structure lead to changes in vertical and horizontal light heterogeneity during tropical forest succession.
3. We described successional patterns in light using a chronosequence approach in which we compared 14 Mexican secondary forest stands that differ in age (8–32 years) since agricultural abandonment. For each stand, we measured vertical light profiles in 16 grid cells, and structural parameters (diameter at breast height, height and crown dimensions) for each tree.
4. During succession, we found a rapid increase in stand size (basal area, crown area and length) and stand differentiation (i.e. a gradual leaf distribution along the forest profile), which leads to fast changes in light conditions and more light heterogeneity. The inflection points of the vertical light gradient (i.e. the absolute height at which 50% relative light intensity is attained) rapidly moved towards higher heights in the first 20 years, indicating that larger amounts of light are intercepted by canopy trees. Light attenuation rate (i.e. the rate of light extinction) decreased during succession due to slower accumulation of the crown area with height. Understorey light intensity and heterogeneity slightly decreased during succession because of an increase in crown size and a decrease in lateral gap frequency. Understorey relative light intensity was 1.56% at 32 years after abandonment.
5. *Synthesis*. During succession, light conditions changed linearly, which should lead to a continuous and constant replacement of species. Especially in later successional stages, stronger vertical light gradients can limit the regeneration of light‐demanding pioneer species and increase the proportion of shade‐tolerant late‐successional species under the canopy. These changes in light conditions were largely driven by the successional changes in forest structure, as basal area strongly determined the height where most light is absorbed, whereas crown area, and to a lesser extent crown length, determined light distribution.

## Graphical abstract

Secondary forest succession is driven by changes in forest structure and light environment, but few studies have quantified both factors. During succession in a Mexican tropical rainforest, light in the understory became more homogeneously distributed, whereas vertical light heterogeneity increased. Stand basal area determined the height where most light is absorbed, whereas crown architecture determined horizontal and vertical light distribution.

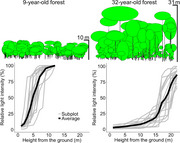

## 1 INTRODUCTION

### 1.1 Justification; the importance of light and the niche of the paper

Light is a key resource for plants, affecting their recruitment, growth and survival (Chen et al., 2014). Therefore, light influences when, where and which plant species can regenerate (Jin et al., 2018; Uriarte et al., 2018). Light is an especially limiting resource in closed forests, and as a result, tree species partition along spatial and temporal gradients in light availability, thus contributing to species coexistence (Kitajima & Poorter, 2008; Kohyama & Takada, 2012). In tropical rain forests, a tall evergreen canopy strongly depletes light availability and creates strong vertical light gradients (Yoda, 1974), which steer the course of secondary succession through one‐sided light competition, where taller individuals can pre‐empt light and suppress the growth of smaller individuals (Finegan, 1996; Schwinning & Weiner, 1998). Although it is well established that light availability decreases during succession due to increasing forest biomass and tree height (Brown & Parker, 1994; Fauset et al., 2017), we still lack an understanding when during succession and where in the canopy the largest changes in light occur, what are the absolute light levels, and how this changes over time. This information is needed to quantitatively underpin and assess hypotheses in forest ecology on light competition, environmental filtering, niche partitioning and species replacement during succession. Here we analyse a Mexican rainforest, how light availability and spatial light heterogeneity change during succession, and how these are driven by concomitant forest structure changes.

### 1.2 The vertical light gradient

From the forest canopy to the forest floor, irradiance shows a marked exponential decline, as light is intercepted by successive leaf layers (Kitajima et al., 2005; Yoshimura et al., 2008). This vertical light gradient has a strong effect on light competition among trees and other plant growth forms, and thus increasing size inequality (i.e. stand differentiation) and mortality risk of suppressed individuals (Weiner, 1990; Yoda et al., 1963), a process called stand thinning. This vertical light gradient is also crucial for species coexistence, as species partition the vertical light gradient by attaining different maximal sizes (Kohyama, 1993; Laurans et al., 2014).

According to the theoretical and modelling studies, vertical light heterogeneity is known to be primarily determined by forest structural attributes, that is, the height distribution of trees, the size (i.e. length and width) of their crowns, and the distribution and density of foliage within crowns (Binkley et al., 2013; Stark et al., 2012). Because vertical light profiles have rarely been measured in forests due to the technical difficulty, they have been estimated by the leaf distributions along the forest profile and mainly inferred from Beer's law (Monsi & Saeki, 1953; Perot et al., 2017) which was developed for herbaceous communities. This law assumes that light intensity is attenuated in a log‐linear way with the cumulative leaf area from the top of the forest canopy down to the ground. Yet, in forests, the vertical light profiles cannot be easily approximated using this model because forests have a more complex canopy structure than herbaceous communities. Therefore, we here quantified the vertical light profile using direct measurements and reveal how forest structural attributes determine the vertical light heterogeneity in secondary tropical rainforests.

### 1.3 The horizontal light gradient

Apart from the ubiquitous vertical light profiles, horizontal light heterogeneity can be large due to the occurrence of vertical and lateral canopy gaps of different sizes, depths, and shapes (Montgomery & Chazdon, 2001), the foliage distribution of the canopy tree species (Binkley et al., 2013) and understorey plants (e.g. palm species; Montgomery, 2004). Horizontal light heterogeneity in the understorey is important as it affects forest regeneration processes (e.g. seed germination, recruitment, growth, and survival of seedlings and saplings; van der Meer et al., 1998) and facilitates species coexistence and, hence, species diversity through light niche partitioning (Jin et al., 2018; Kumar et al., 2018).

### 1.4 Successional gradients in light availability

During secondary succession, community structure and complexity increase rapidly, with profound consequences for the amount of light and its spatial heterogeneity within forests (Brown & Parker, 1994; Neufeld & Young, 2014). Light availability in the understorey, for example, strongly decreases during succession due to an increase in forest height and stand size (i.e. basal area, and crown area and length; Lebrija‐Trejos et al., 2010, 2011). This successional decline in understorey light availability steers the course of succession, as regenerating light‐demanding early successional species are gradually replaced by more shade‐tolerant late‐successional species. For example, gaps formed by early‐successional species that die early in succession provide the opportunity for an understorey re‐initiation phase in succession (Oliver, 1980). This phase provides opportunities for a second wave of regeneration of late‐successional species and facilitates the growth of existing late‐successional species in the understorey, and hence leads to the replacement from early‐successional species to late‐successional species (Finegan, 1996; Martínez‐Ramos et al., 1989). Although many studies have assessed understorey light environment during succession, surprisingly little is known about how vertical and horizontal light heterogeneity change with forest development during secondary succession (but see Brown & Parker, 1994; Lebrija‐Trejos et al., 2011). To our knowledge, this is one of the first comprehensive studies that evaluates successional changes in light heterogeneity in tropical secondary forests. Successional changes in light availability might be fundamentally different for tropical forests compared to temperate forests. In tropical forests, early successional stands have, compared to temperate forests, a larger number of species, a complex structure (many heights versus two layers), pioneer tree species are shorter lived resulting in higher turnover and early gap dynamics, and pioneers have a faster height growth resulting in a faster pace of succession, with large consequences for successional changes in light environments (Chazdon et al., 2007). Hence, patterns and processes that apply to temperate forest succession do not necessarily apply to tropical forest succession.

### 1.5 Aim, questions and hypotheses

This study aims to evaluate how changes in forest structural attributes lead to changes in vertical and horizontal light heterogeneity during tropical forest succession. We addressed two questions: (a) How, when and where do forest structural attributes and the vertical and horizontal light heterogeneity change during succession? We predict that forest height and stand size increase during early stage of secondary succession, which is followed by an increase in light interception at the top of the stands, and hence increases vertical light heterogeneity rapidly and decreases the horizontal light heterogeneity in the understorey. (b) Which, when and how do forest structural attributes shape vertical and horizontal light heterogeneity? We hypothesize that during succession (a) vertical light heterogeneity increases because of an increase in basal area of larger trees as basal area scales strongly with leaf area (Shinozaki et al., 1964), and thus the higher inequality of basal area at later successional stages leads to a large amount of light interception in the top of the stand and less light transmission to the forest floor and (b) horizontal light heterogeneity in the understorey decreases because of more homogeneous crown distributions within the stand leading to a lower frequency of vertical and lateral gaps.

## 2 MATERIALS AND METHODS

### 2.1 Study site

This study was conducted in Loma Bonita (16°04′N; 90°55′W), Marqués de Comillas, southeast Mexico. The climate is warm‐humid with a mean annual temperature of 24°C and an annual rainfall of around 3,000 mm, with a dry period (less than 100 mm/month) from February to April (Martínez‐Ramos et al., 2009). The study sites are located in low hills (an area with small hills and valleys), where sandy and clay soils of low pH (<5.5) predominate (Navarrete‐Segueda et al., 2018). The original vegetation consists mainly of lowland tropical rainforests and semi‐deciduous forests (Ibarra‐Manríquez & Martínez‐Ramos, 2002).

### 2.2 Field survey

To analyse successional trends in forest structure and light environment, we used a chronosequence approach, in which we compared 14 secondary forest plots with different ages since agricultural abandonment. The plots consisted of natural regeneration developed on abandoned cornfields and with a fallow age of 8–32 years at the time of measurement (cf. van Breugel et al., 2006). Each plot's size in this study was 40 × 10 m and divided into 16 subplots of 5 × 5 m. Each individual with more than 1 cm stem diameter at breast height (DBH) was mapped and identified to species, and its DBH and height were measured. Height was measured with a telescope rod or a range finder. In February 2019, for each individual, the crown base (i.e. the distance between the ground and the lowest live branches in the crown of a tree) and crown width were measured in two orthogonal cardinal directions (north‐south and east‐west).

The vertical light profile in the forests was measured using a Photosynthetic Photon Flux Density (PPFD) sensor (DEFI2‐L, JFE Advantech Co., Ltd) attached to a 20 m telescopic carbon rod (Taketani Trading Co.). Instantaneous measurements were taken under overcast sky conditions to represent the average light environment during the growing season in the studied site without the confounding influence of direct sunlight. For example, Parker et al. (2002) found that vertical light profiles measured under overcast sky conditions were smoother than those under clear sky conditions. Furthermore, diffuse light under overcast sky conditions can penetrate deeper into forest canopies than direct light, and thus more influenced light partitioning among tree individuals and be more determined by forest structure (Alton et al., 2007; Zhang et al., 2011). Therefore, although we acknowledge the importance of direct light and sunflecks on plant performance, we focus on diffuse light (Fauset et al., 2017; Marler, 2017). At the centre of each of the 16 subplots, irradiance was measured at height intervals of 1 m, from 1 to 22 m height (i.e. 22 light environments per subplot). At each height, PPFD was measured for 5 s and averaged. To standardize the light measurements for temporal variation in light intensity across different measurements, relative light intensity (in %) was calculated for each height. For this, the irradiance at a given height was divided by irradiance above the canopy or by the irradiance that was simultaneously measured in an open area near the plot and multiplied by 100.

### 2.3 Vertical and horizontal light heterogeneity

To describe the vertical light profile in the stands, we related the relative light intensity to the height in the stand by fitting for each subplot a logistic sigmoid curve (Equation 1):

(1) Y=100/[1+exp{(‐a×(x‐b))}],

where *Y* is the relative light intensity (%), *x* is the height (m), *a* is the slope at the inflection point and *b* is the height of inflection point (i.e. where 50% of relative light intensity is attained). We used a logistic sigmoid curve because the predicted line of the regression curve is constrained between 0% and 100%, thus avoiding unrealistic values (i.e. light levels above 100% or below 0%). More importantly, it provides estimates of two important ecological parameters; the light attenuation rate, which is described by *a,* the slope at the inflection point, and the vertical position in the stand where the strongest light attenuation occurs, which is described by *b*, the height of the inflection point (HIP). During secondary succession, stands increase rapidly in height. To compare the HIP among stands with different ages and heights, we also calculated the relative height of inflection point (in %), dividing HIP by the maximum canopy height of the whole plot and multiplied by 100. We used the maximum height of the whole plot because the vertical light gradient is also strongly affected by neighbouring trees rooted in different subplots.

To quantify the horizontal variation in light availability in each plot, we calculated each of the 22 reference heights (i.e. from 1 to 22 m) the standard deviation of relative light intensity, considering light records from the 16 subplots at each reference height. We used the absolute standard deviation rather than the coefficient of variation, as from an ecological point of view, plants respond to absolute light variation. To infer how horizontal light heterogeneity in understorey may affect the regeneration of tree species with different light requirements, we compared light heterogeneity at 1 m height across the different‐aged stands, our nearest measurement to the forest floor where seeds germinate and plants recruit. To determine the vertical position in the stand where the strongest horizontal light heterogeneity occurs, we examined the absolute heights of the highest horizontal light heterogeneity per plot. To compare the heights with the highest horizontal light heterogeneity among stands with different ages and canopy heights, we relativize this height. For each plot, the relative height of the highest horizontal light heterogeneity was calculated as the absolute height of the highest horizontal light heterogeneity divided by the maximum canopy height of the whole plot.

### 2.4 Forest structural attributes

To quantify vertical forest structural attributes, we first assumed multi‐stemmed trees as one single tree using the square root of the sum of the squares of each individual stem diameter as DBH of multi‐stemmed trees (Awang et al., 1994). Then, we calculated tree basal area as 0.25 × *π* × DBH^2^, tree crown area as 0.25 × π × *d*1 × *d*2, in which *d*1 and *d*2 are the two crown diameter measurements, and tree crown length as the height of the tree minus the height of the crown base per tree (Poorter et al., 2006). We analysed the height distribution of forest structural variables in each stand, by plotting the cumulative value of each forest structural attribute (i.e. basal area, height, crown area and crown length of trees) against cumulative tree height (i.e. by ranking individuals in increasing order of tree height). To summarize these profiles, we fitted for each forest attribute and plotted the same logistic sigmoid model (Equation 1) on each cumulative curve of forest structural attributes to obtain the slope of the inflection point (i.e. how steeply forest structural attributes changes with height) and HIP (i.e. at what height level do most forest structural changes occur) for each attribute and plot.

To evaluate plot‐level forest structural attributes, we calculated the total forest structural attributes per plot; we counted numbers of trees with DBH more than 1cm and summed basal area, crown area and crown length per plot. To assess the horizontal variation in each of these structural attributes, we calculated for every one of the 16 subplots the mean value and *SD* of each attribute, and then the coefficient of variation as 100 × *SD*/mean. We used the coefficient of variation rather than the absolute standard deviation, as relative variation is less sensitive to differences in the mean value of each structural attribute among different plots, and hence can be a more precise predictor of the horizontal forest structural heterogeneity among different‐aged stands.

### 2.5 Data analysis and statistical analysis

All data analyses were conducted using R Studio (version 1.1.453; R Foundation for Statistical Computing). To visualize the successional changes in forest structure, we plotted plot‐level forest structural attributes and coefficient of variation of forest structural attributes against the stand age of the plot. To describe successional patterns in light attributes, we plotted vertical light heterogeneity (i.e. light attenuation rate and absolute and relative HIP of relative light intensity), horizontal light heterogeneity (absolute and relative height of the highest horizontal heterogeneity, and horizontal light heterogeneity at 1 m above‐ground), and relative light intensity at 1 m above the ground against the stand age of the plot. Then, we assessed these relationships with linear mixed models, with plots as random intercept using the lme4 package in R (Kuznetsova et al., 2017). In case these models did not meet the parametric assumptions, we used the nonparametric Mann–Kendall trend procedure instead, followed by Sen's protocol to obtain the slope and intercept of its regression line, using the modifiedmk package in R (Patakamuri et al., 2020).

To examine which forest structural attributes shape light heterogeneity, we performed two types of analyses. First, we used a linear mixed model or the nonparametric Mann–Kendall procedure to evaluate the effects of each structural attribute on three measures of light heterogeneity (light attenuation rate, HIP of relative light intensity and horizontal light heterogeneity at 1 m) and relative light intensity at 1 m. Second, to show the direct and indirect effects of successional age on forest structural attributes and light heterogeneity, path models were created using the psem function of the piecewisesem package (Lefcheck, 2016). One path model was created for each metric of the four measures of light heterogeneity. Because forest structural attributes were strongly correlated with each other and to avoid collinearity, we only selected two structural attributes for the path model. We first selected the structural attribute that showed the strongest correlation with the light descriptor, and second selected the structural attribute that had the weakest correlated with the first structural attribute based on Pearson pairwise correlations (Figure S1; Table S1). For light attenuation rate, all the variables were log‐transformed to improve normality and homoscedasticity (the original names of the variables are used in the text and figures).

## 3 RESULTS

### 3.1 Vertical and horizontal light distribution during succession

**FIGURE 1:**
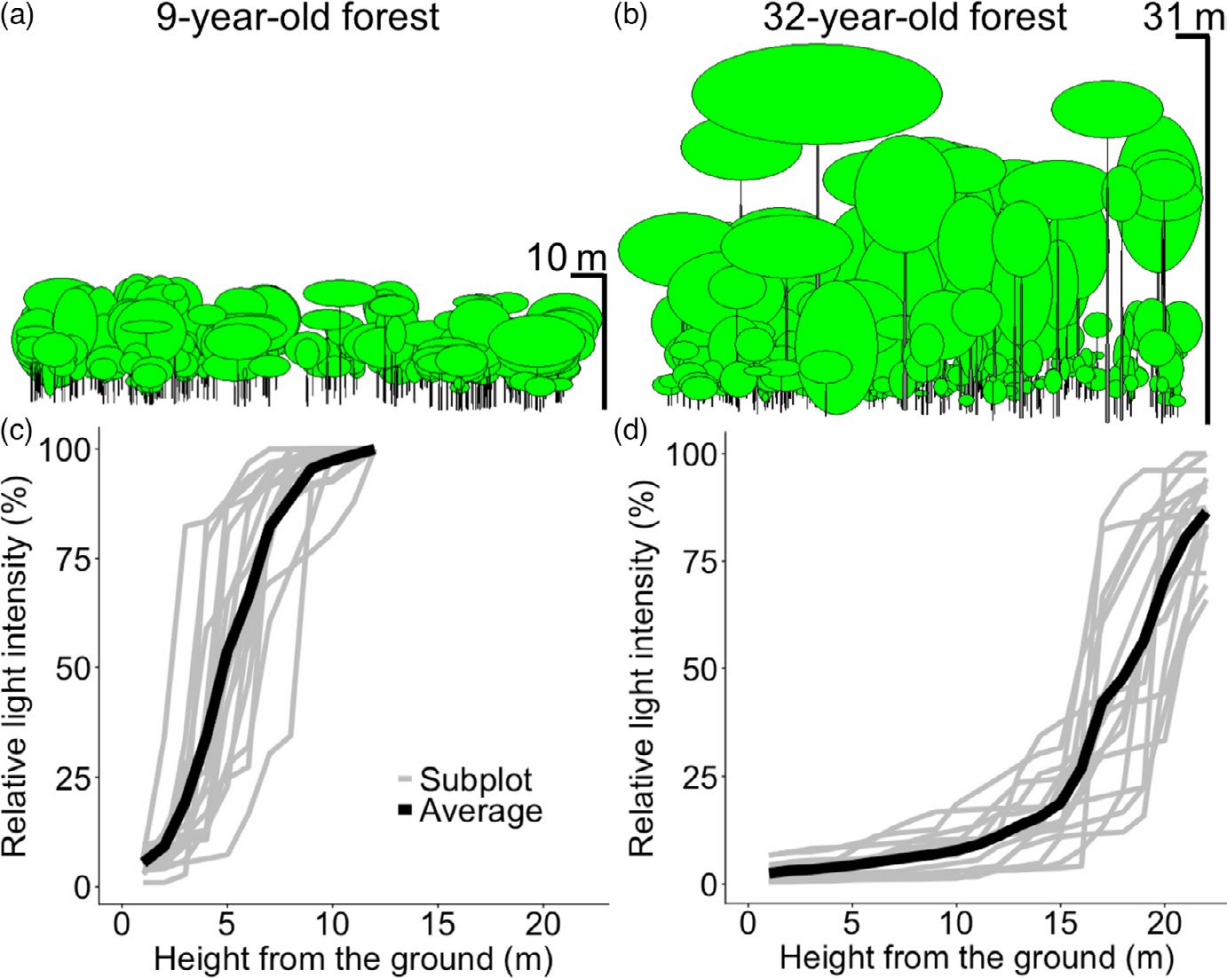
Forest structural changes during secondary succession in Marqués de Comillas, southeastern Mexico. Panels in (a) and (b) show three‐dimensional images of forest structure and those in (c) and (d) corresponding vertical light profiles for a stand of 9 years old (left panels) and a stand of 32 years old (right panels). Height, crown size and tree position are drawn based on actual measurements using Forest Window (2.24). Grey lines represent the light profiles of each of the 16 subplots, and black lines represent the average light profile across the 16 subplots

Forest structure developed both vertically and horizontally during secondary succession as the canopy became taller and canopy trees tended to have longer and wider crowns at later successional stages (Figure 1a,b; Figures S2 and S3).

**FIGURE 2:**
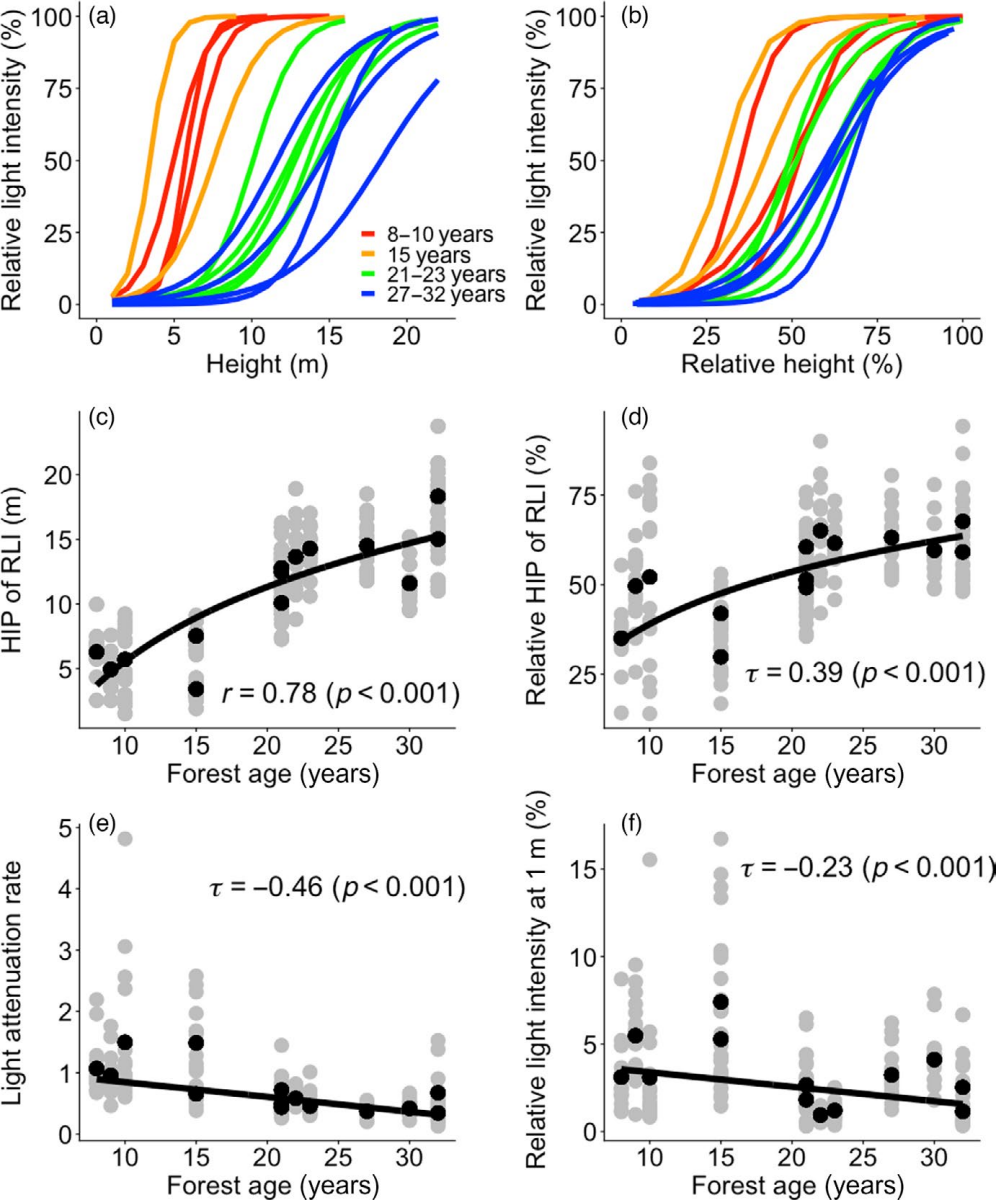
Mean vertical light profile based on sigmoidal fitted curves against (a) the absolute height (m) and (b) the relative height compared to the maximum canopy height per plot (%). Light profiles are shown for 14 plots that differ in forest age since field abandonment (as indicated by different colours). (c) Absolute and (d) relative height of inflection point (HIP) of relative light intensity (RLI), (e) light attenuation rate and (f) RLI at 1 m versus forest age. Grey dots represent the value of each of the 16 subplots, and black dots represent the average value across the 16 subplots per plot. The results of the regression line (black line) and coefficient of determination (*r*: Pearson correlation coefficient and *τ*: Kendall's Tau) are shown. Pearson correlation coefficient was obtained using linear mixed model setting plots as a random factor. Kendall's Tau was obtained using the nonparametric Mann–Kendall trend procedure, and the slope and intercept of its regression line were obtained using Sen's protocol. Light attenuation rate is the slope of light extinction at the inflection point, and HIP is the height of 50% RLI based on sigmoidal fitted curves per plot. Regression lines are (c) *y* = 8.31 log(*x*) − 13.56; (d) *y* = 21.11 log(*x*) − 9.62; (e) *y* = −0.024*x* + 1.09; (f) *y* = −0.083*x* + 4.22

Light distribution was highly heterogeneous, both vertically and horizontally, within the same forest stand (Figure 1c,d; Figure S4) and also among different‐aged stands (Figure 2a,b). During succession, the average absolute height of the inflection point of light intensity (i.e. the height in the stand where relative light intensity is 50%; HIP of light intensity) increased from 3.4 to 18.3 m (Figure 2c), and the average relative HIP increased from 29.8% to 67.6% (Figure 2d). This means that in later successional stages 50% of light is absorbed at taller heights (in absolute and relative terms). Absolute and relative HIP of light intensity changed drastically during early stages of succession as it increased with 0.56 m and 1.46% per year during the first 20 years of succession, but increased only with 0.34 m and 0.86% per year after 20 years of succession. The light attenuation rate (i.e. the slope of light extinction at the inflection point) decreased gradually and linearly with forest age, indicating that light is more gradually absorbed when it passes through the canopy at later successional stages (Figure 2e). Average relative light intensity at 1 m above‐ground decreased gradually and linearly with forest age from 3.56% at 8 years after abandonment, to 1.56% after 32 years (Figure 2f; Table S2), although an intermittent peak of 7.40% was found at 15 years due to local gap formation.

**FIGURE 3:**
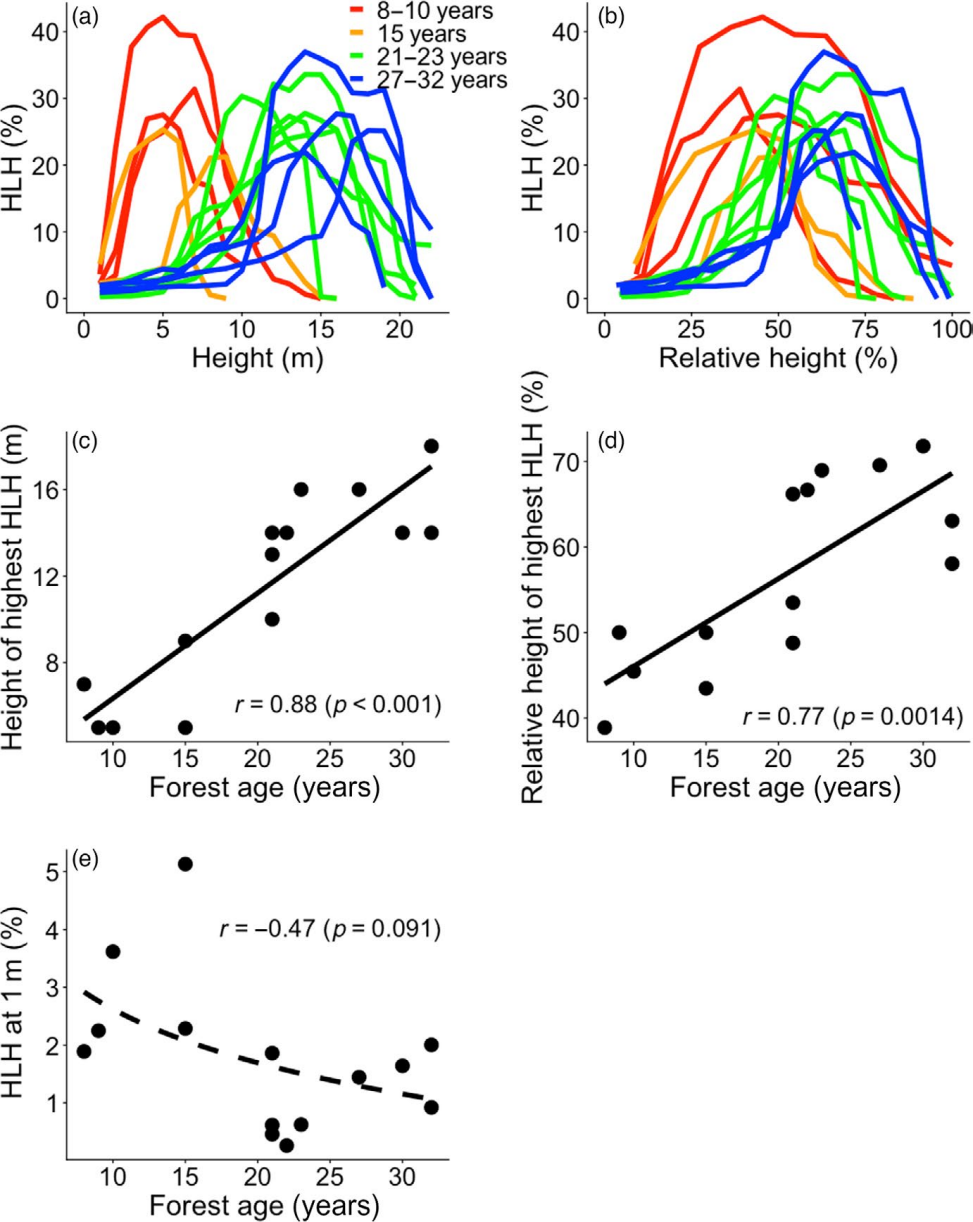
Horizontal light heterogeneity (HLH, expressed as the standard deviation in relative light intensity across 16 subplots at the same height) versus (a) the absolute height (m) and (b) the relative height compared to the maximum canopy height per plot (%). Curves are shown for 14 plots that differ in forest age since field abandonment (as indicated by different colours). In (c) and (d) are shown the changes in the absolute and relative height with the highest HLH versus forest age. The change of HLH at 1 m with forest age is shown in (e). The results of the regression line (black line) and the coefficient of determination (*r*) are shown. Regression lines are (c) *y* = 0.49*x* + 1.48; (d) *y* = 1.03*x* + 35.78; (e) *y* = −1.34 log(*x*) + 5.70

The horizontal light distribution varied strongly within a forest stand and among differently aged stands (Figure 3a,b). Horizontal light heterogeneity (i.e. the standard deviation of relative light intensity across 16 subplots) at a given height showed a hump‐shaped pattern with absolute and relative height from the ground and peaked around 40%–50% of canopy height for early successional stages and around 75% for later successional stages (Figure 3a,b). The coefficient of variation of relative light intensity across 16 subplots also showed similar patterns. That is, it showed hump‐shaped patterns with absolute and relative height from the ground (Figure S5). Therefore, the absolute and relative height at which we found the largest horizontal light heterogeneity significantly increased with forest age (Figure 3c,d). Horizontal light heterogeneity at 1 m decreased slightly but not significantly with forest age (Figure 3e).

### 3.2 Vertical and horizontal variation in forest structural attributes during succession

**FIGURE 4:**
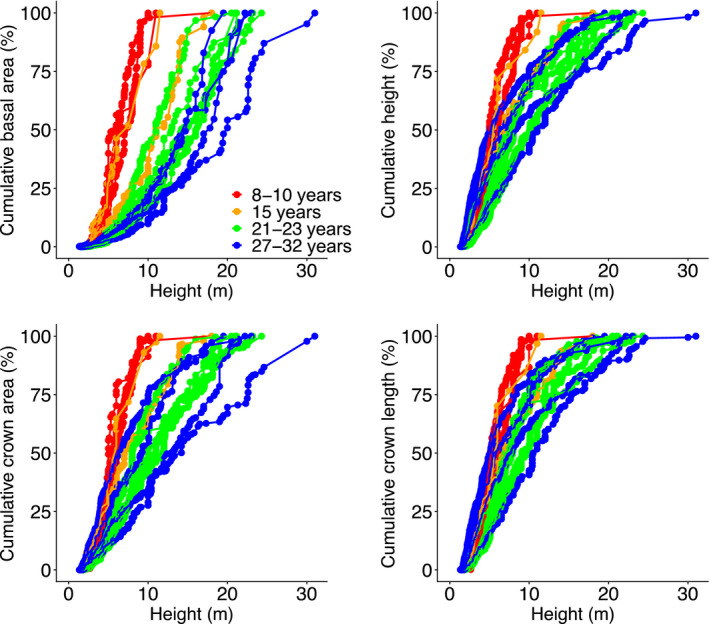
Changes in forest structural attributes during succession. Cumulative value of (a) basal area, (b) height, (c) crown area and (d) crown length against cumulative tree height (i.e. by ranking individuals in increasing order of tree height) for 14 plots that differ in forest age since field abandonment (as indicated by different colours). Each dot represents one individual

We here present vertical patternsin forest structure to assess whether they may underlie vertical patterns in light extinction. All cumulative forest structural attributes increased with height within the forest stand, and this pattern changed during succession (Figure 4). In the early stages of succession, all cumulative structural attributes increased steeply and linearly with height from the ground. In contrast, in later successional stages, the cumulative basal area increased exponentially with height, and the other structural attributes increased more gradually and linearly with height (Figure 4). This means that in later ages of succession, larger trees play a disproportionally important role in determining the plot‐level basal area.

Forest structural attributes changed significantly with forest age, indicating the vertical development of the forest with a larger amount of foliage as succession advances (Figure S3). In contrast, the horizontal heterogeneity of most forest structural attributes did not vary systematically with forest age (Figure S6).

### 3.3 Spatial variation in light is driven by forest structural attributes

**FIGURE 5:**
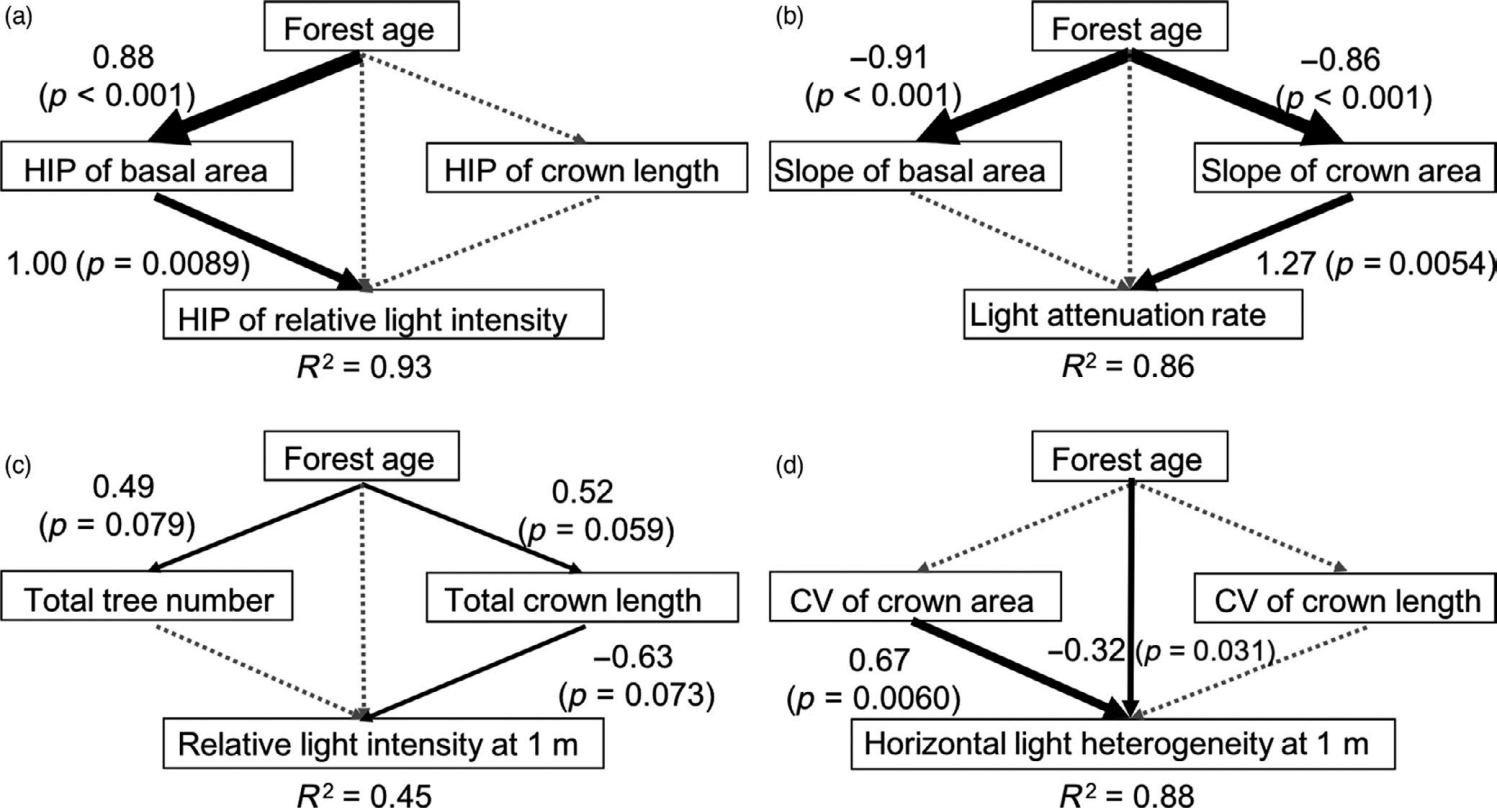
Path models of the standardized effects of forest age on (a) the height of inflection point (HIP) of basal area and crown length, and their effect on the mean HIP of relative light intensity (i.e. the height of 50% cumulative basal area and crown length and relative light intensity), (b) the slope of cumulative basal area and crown area, and their effect on the mean light attenuation rate (i.e. the rate of light extinction), (c) total tree number and total crown length, and their effect on mean relative light intensity at 1 m and (d) coefficient of variation (CV) of forest structural attributes, and their effect on horizontal light heterogeneity at 1 m (expressed as the standard deviation in relative light intensity across 16 subplots at 1 m). Black solid lines indicate significant effects; the values shown next to the arrows are standardized regression coefficients. Grey dotted lines show the full models tested by path models. The thickness of the lines reflects the strength of the effect. The coefficient of determination (*R* ^2^) is shown for the final response variable only. In (b), all the variables were log‐transformed to improve normality and homoscedasticity, but the original names are kept for simplicity

**FIGURE 6:**
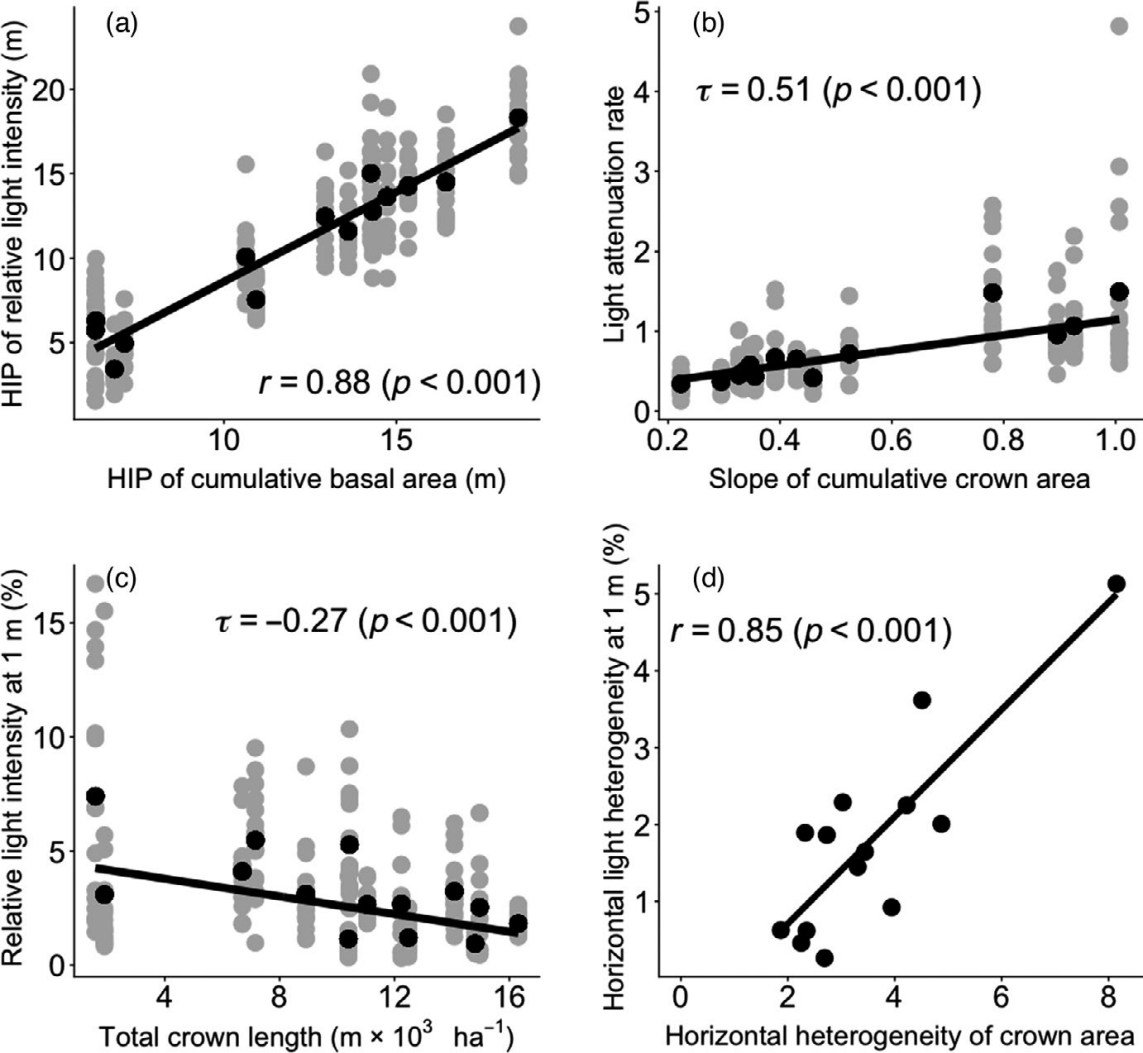
Effects of forest structural attributes on light heterogeneity. (a) Height of inflection point (HIP) of relative light intensity versus HIP of cumulative basal area, (b) light attenuation rate (i.e. the slope of light extinction) versus the slope of the cumulative crown area, (c) relative light intensity at 1 m versus total crown length per plot and (d) horizontal light heterogeneity at 1 m (expressed as the standard deviation in relative light intensity across 16 subplots at 1 m) versus horizontal heterogeneity of crown area (expressed as the coefficient of variation (%) in the crown area). In (a, b and c), grey dots represent the value of each of the 16 subplots, and black dots represent the average value across the 16 subplots per plot. The results of a regression line (black line) and coefficient of determination (*r*: Pearson correlation coefficient and *τ*: Kendall's Tau) are shown. Pearson correlation coefficient was obtained using linear mixed model setting plots as a random factor. Kendall's Tau was obtained using the nonparametric Mann–Kendall trend procedure, and the slope and intercept of its regression line were obtained using Sen's protocol. HIP of relative light intensity and the cumulative basal area is the height of inflection point, and light attenuation rate and slope of cumulative crown area are the slopes at the inflection point, as obtained by fitted sigmoidal curves per plot. Regression lines are (a) *y* = 1.07*x* − 2.03; (b) *y* = 0.96*x* + 0.19; (c) *y* = −0.19*x* + 4.56; (d) *y* = 0.70*x* − 0.69

We used path models to assess how forest structure changes with forest age, and how forest structure, in turn, affects light distribution (Figure 5). Figure 5 shows the results of the path model, and Figure 6 shows the corresponding bivariate relationships. An increase in forest age led to an increase in forest height at which half of the basal area is attained (i.e. HIP of basal area, Figure 5a) and a slower and more gradual accumulation of crown area with height (‘slope of crown area’, Figure 5b). Surprisingly, forest age had weak positive effects on total tree number and total crown length, and did not significantly affect horizontal heterogeneity of forest structural attributes (Figure 5c,d). The HIP of the basal area had a strong positive effect on the HIP of RLI (*β* = 1.00, Figures 5a and 6a), indicating that when basal area accumulates at taller heights, that light is also intercepted at taller heights. A faster accumulation of crown area with height (‘slope of crown area’) led to a strong increase in light attenuation rate (*β* = 1.27, Figures 5b and 6b). Longer total crown length per plot decreased relative light intensity at 1 m (*β* = −0.63, Figures 5c and 6c). A larger horizontal heterogeneity in crown area resulted in a larger horizontal light heterogeneity at 1 m height above the forest floor (*β* = 0.67, Figures 5d and 6d), while an increase in forest age led to a decrease in horizontal light heterogeneity at 1 m (*β* = −0.32, Figure 5d).

## 4 DISCUSSION

We evaluated how successional changes in the structure of secondary tropical forests lead to changes in vertical and horizontal light heterogeneity. During succession, stand size (basal area, crown area and length) and stand differentiation (i.e. a gradual leaf distribution along the forest profile) increased, which resulted in a decrease in light attenuation rate in the canopy and a decrease in light intensity and heterogeneity in the understorey. Here, we first discuss general successional patterns, then how forest structure affects irradiance and finally their implications for light competition and species turnover during succession.

### 4.1 Successional changes in light heterogeneity during stand development

As expected, we found that succession leads to a rapid increase in stand size during the first 30 years, and, hence, to an increase in the vertical light heterogeneity (i.e. slower light attenuation rate and higher absolute and relative HIP of RLI, Figure 2c,d,e), and a reduction in the horizontal light heterogeneity in the understorey. Successional changes in light attenuation rate in the studied tropical forest are faster compared to those found for temperate forests (Brown & Parker, 1994).

Our observed successional changes in light profiles can be associated with the four stand development phases of Oliver (1980): stand initiation, stand exclusion, understorey re‐initiation and old‐growth phase. First, in the initiation phase, even‐sized canopy trees (Figure 4) result in dense foliage, and thus strong light attenuation (Figure 2e). Second, in the stand exclusion phase, the fast growth of pioneers, strong light competition and stand thinning lead to stand differentiation, and hence gradual leaf distribution along the forest profile (Figure 4), which results in a more gradual light attenuation (Figure 2e). In the third phase, the mortality of short‐lived pioneers leads to the understorey re‐initiation phase. For instance, short‐lived pioneer trees such as *Trema micrantha* start to die as early as after 15 years (van Breugel et al., 2007), thus creating canopy gaps, which result in high light intensity and horizontal light heterogeneity at the forest floor (Figures 2f and 3e), thus providing regeneration opportunities for small gap specialists. Finally, in the old‐growth phase, larger long‐lived pioneers have longer and wider crowns leading to a larger amount of light interception at absolutely and relatively higher height, which results in the higher HIP of RLI (Figure 2c,d) and lower light levels in the understorey (Figure 2f). Since a larger amount of light is intercepted by canopy trees, the presence and absence of canopy trees and their leaves results in a huge difference in the light availability from place to place, which leads to stronger horizontal light heterogeneity at higher height (Figure 3c,d).

Although similar stand development patterns and concomitant light changes occur in forests worldwide, the ideal growing conditions in tropical rain forests (a warm and wet climate year‐round) compared to temperate forest speeds up stand development, leading to faster rate of light changes and successional trajectories. The fast growth of tropical pioneer trees which grow up to 5 m per year in this studied site compared to, for example, 1 m per year for the temperate pioneers (T. Matsuo, unpubl. data) leads to a rapid decline in light availability at the forest floor (as low as 1.12% after 8 years, Table S2). Different life histories (short‐lived tropical pioneers live for 5–20 years compared to more than 50 years for temperate pioneers; Loehle, 1988; van Breugel et al., 2007) lead to rapid, endogenously driven gap formation and understorey re‐initiation (after 1.5 decades instead of after 5 decades). This high turnover rate provides more regeneration opportunities for different species and increases biodiversity (after 3 decades, more than 50 species per 400 m^2^ are found at our studied site, compared to less than 25 species in temperate forests; T. Matsuo, unpubl. data) and leads to a more uneven aged and structurally complex stand structure.

Hence, secondary succession of wet tropical forest reflects the stand development phases of Oliver, but the fast speed of forest development leads to faster and stronger changes in light conditions, more light heterogeneity and different successional pathways compared to temperate forests.

### 4.2 Structural development drives successional patterns in irradiance

During succession, the rapid increase in forest height and stand size (because canopy trees grow taller, thicker and have larger crowns; Figure 1a,b; Figure S3), and the competition driven by the increase in size inequality among tree individuals leads to stand differentiation (Zhang & Chen, 2015; van Breugel et al., 2012). This structural development had strong implications for successional changes in forest light environments.

An increase in cumulative basal area during succession led to an increase in the absolute HIP of RLI, most likely because trees with the larger basal area are taller and because the amount of foliage within the crown scales closely with tree basal area (Enquist, 2002; Shinozaki et al., 1964). Therefore, at the older successional ages, the foliage is more concentrated at taller heights, resulting in a larger proportion of intercepted light in the top canopy of the stand (Figures 5a and 6a).

Crown area and length are important for the packing and distribution of leaves, and hence, the light distribution in the forest. An increase in (overlapping) crown area during succession leads to a more rapid canopy closure and a stronger light attenuation (Figure 5b), while the increase in horizontal heterogeneity in the crown area increases lateral gaps, and hence horizontal light heterogeneity at the forest floor (Figure 5d; Montgomery & Chazdon, 2001). Longer crowns lead to more leaf stacking, a higher leaf area index and more light interception (Kitajima et al., 2005) and therefore to a lower light availability in the understorey (Figure 5c; Lebrija‐Trejos et al., 2010; Whitmore et al., 1993).

Surprisingly, light attenuation rate was more driven by the crown area than by basal area (Figure 5b). Basal area is easily measured and scales strongly with leaf area (Lehnebach et al., 2018; Shinozaki et al., 1964), and is therefore frequently used to model light extinction and light competition in forest stands (Rozendaal et al., 2020; Zhang et al., 2015). Perhaps the difference is that we evaluated light attenuation rates at the height of the inflection point. Hence, although the total stand basal area scales with stand leaf area index and the length of the light gradient, our light attenuation rate indicates the slope where most light extinction occurs. And that occurs with a rapid accumulation of crown area (i.e. the slope of the crown area) and, hence, leaves.

In sum, basal area determines the height in the stand where most light is absorbed, whereas crown dimensions, especially crown area, determine the light distribution (i.e. light attenuation rate, and light level and horizontal light heterogeneity in the understorey). To effectively model light extinction and light competition, forest models should go beyond basal area, and also take the role of the cumulative crown area into account.

### 4.3 Implications for light competition

The height of inflection points for light interception showed a logarithmic relationship over time (Figure 2c), indicating that early in succession there is a premium on fast height growth, whereas later in succession fast height growth becomes less important. A steeper light attenuation rate intensifies light competition as a small increase in height results in a disproportional increase in light availability. In early successional ages, the light attenuation rate was highest (Figure 2e), indicating that a competing tree can increase the light interception proportionally more for given height growth. This puts a premium on the faster vertical height growth of light‐demanding pioneer species. An inherently fast growth rate (Poorter et al., 2006; Sterck, Poorter, et al., 2006) and adaptive tree architecture (Sterck & Bongers, 2001; Sterck, van Gelder, et al., 2006) allow pioneer species to out‐compete neighbouring trees, and hence results in faster vertical development and stand differentiation during early succession.

In later successional ages, the majority of light is intercepted at taller heights (Figure 2c,d), which leads to less light availability under the canopy layer. Such a reduction imposes most of the smaller individuals below the canopy, or in the understorey, to intercept sufficient light energy for their growth. Reduced light transmission under the main canopy layer promotes, therefore, stand thinning of sub‐canopy and understorey individuals (McDowell et al., 2018).

Therefore, in early successional stages, an intense light competition due to a steeper vertical light gradient puts a premium on fast height growth of pioneer species, and hence can lead to faster vertical development and stand differentiation. However, in later successional stages, reduced light availability under the canopy layer due to larger canopy individuals can inhibit individuals under canopy to intercept sufficient light for their performance, and hence can promote stand thinning under the canopy layer.

### 4.4 Implications for species turnover and succession

Successional changes in vertical light profiles and light competition strongly affect the course of succession (Horn, 1974). Interestingly, with the exception of HIP all measurements of light distribution changed linearly over time (Figures 2d,e,f and 3c,d), indicating that during succession there are continuous shifts in light distribution with large consequences for species performance and replacement. At early successional ages, we found high horizontal light heterogeneity below 50% of the canopy height (Figure 3c,d) and in the understorey (relative light intensity at 1 m ranges from 0.082% to 15.50% Figures 2f and 3e; Table S2). Such horizontal light variation allows for differential recruitment, survival and growth rate of trees from place to place, and hence facilitates opportunities for light resource partitioning among different species (Kitajima & Poorter, 2008).

At older ages of succession, we found high HIP of relative light intensity and horizontal light heterogeneity above 50% of the canopy height (Figures 2c,d and 3). The larger amount of light interception at the top of the forest stand might limit the survival and growth of pioneer light‐demanding species at the sub‐canopy layer and increase the proportion of later‐successional shade‐tolerant species (van Breugel et al., 2007). This stronger vertical light gradient also enhances vertical light partitioning among different tree species (Kohyama & Takada, 2009; Laurans et al., 2014). The larger horizontal light variation at higher height influences predictability of further development and the flowering and fruiting behaviour of trees (Stephenson, 1981).

Once short‐lived pioneers start to die after 7–15 years, create canopy gaps and locally increase light availability in the understorey and steepen the vertical light gradient (Figure 2e,f), there is a potential combination of three successional pathways: (a) intensification of the race for the canopy by short and long‐lived pioneer species, (b) more frequent regeneration in newly formed gaps by small gap specialists or (c) invasion by other life forms. In the first successional pathway, which has a steep light gradient, light‐demanding pioneers grow faster and form the canopy layer and shade‐tolerant species regenerate in the understorey, which leads to an understorey re‐initiation phase. In the second successional pathway, the local creation of small canopy gaps sets back succession to the stand initiation phase (van Breugel et al., 2007), where species with an intermediate light demand or small gap specialists can recruit and grow quickly under well‐lit conditions. In the third successional pathway, invasive species such as lianas or bracken ferns become dominant and hamper tree species regeneration (which may last for several decades in an arrested successional phase), when gaps are extensive and the soils have degraded conditions (Schnitzer et al., 2000; Suazo‐Ortuño et al., 2015).

In sum, linear changes in light conditions during succession lead to a continuous and constant replacement of species. Canopy gaps during early ages of succession due to the death of short‐lived pioneer species can introduce different scenarios depending on the size of the gaps, seed sources and soil quality. In older ages of succession, stronger vertical light gradients can increase the proportion of later‐successional, shade‐tolerant species under canopy layers by limiting the regeneration of pioneer, light‐demanding species, and therefore enhance vertical light partitioning among different tree species.

## 5 CONCLUSIONS

In wet tropical forests, a fast speed of forest development leads to faster changes in light conditions, more light heterogeneity and different successional pathways. We found that, during succession, light conditions reduce linearly, which should lead to a continuous and constant replacement of species. Especially in later successional stages, larger vertical light gradients can limit the regeneration of pioneer light‐demanding species, and hence increase the proportion of late‐successional, shade‐tolerant species under canopy layers. These changes in light conditions are strongly driven by the successional changes in forest structure, as basal area strongly determines the height where most light is absorbed, whereas crown area, and to a lesser extent crown length, determines light distribution. Such complex spatial–temporal gradients in light heterogeneity conform to a basic resource patchwork for photosynthesis, which drives light competition, environmental filtering, niche partitioning and species replacement during secondary succession.

## AUTHORS' CONTRIBUTIONS

T.M. and L.P. developed the idea and led the writing of the manuscript; T.M. performed the fieldwork; M.M.‐R. and F.B. provided the chronosequence system; T.M. performed the data analysis; L.P and M.T.v.d.S. contributed to data analysis; F.B., M.T.v.d.S. and M.M.‐R. provided the comments. All the authors contributed critically to the drafts and gave final approval for publication.

### PEER REVIEW

The peer review history for this article is available at https://publons.com/publon/10.1111/1365‐2745.13680.

## Supporting information

Supplementary Material

## Data Availability

The data on light measures and structural attributes are stored in DANS https://doi.org/10.17026/dans‐zjn‐dn5b (Matsuo et al., 2021).
