# Supplementary material for: Forest structure drives changes in light heterogeneity during tropical secondary forest succession
Source: J Ecol. 2021 May 27;109(8):2871–84. doi: 10.1111/1365-2745.13680 (PMC8453511; doi:10.1111/1365-2745.13680)
Supplement: Supplementary file 1 — Supplementary Material [file JEC-109-2871-s001.docx]

Table S1. Values of Pearson pair-wise correlation among: (a) the mean height of inflection point (HIP) of relative light intensity (RLI) across 16 sub-plots and the cumulative structural attribute per plot, (b) the mean light attenuation rate across 16 sub-plots (i.e., the slope of light extinction at the inflection point) and the slope of cumulative forest structural attributes per plot, (c) the mean RLI at 1m above the ground across 16 sub-plots and forest structural attributes per plot, and (d) standard deviation (SD) of RLI at 1m across 16 sub-plots (i.e., horizontal light heterogeneity at 1m) and coefficient of variation (CV) of forest structural attributes (i.e., horizontal heterogeneity in forest structural attributes) per plot. Significant correlations (p<=0.05) are expressed with bold letters.

| (a) |  |  |  |  |  |
| --- | --- | --- | --- | --- | --- |
| HIP of RLI |  |  |  |  |  |
| HIP of basal area | **0.96** |  |  |  |  |
| HIP of height | **0.79** | **0.84** |  |  |  |
| HIP of crown area | **0.89** | **0.92** | **0.93** |  |  |
| HIP of crown length | **0.70** | **0.77** | **0.99** | **0.88** |  |

| (b) |  |  |  |  |  |
| --- | --- | --- | --- | --- | --- |
| Light attenuation rate |  |  |  |  |  |
| Slope of basal area | **0.74** |  |  |  |  |
| Slope of height | **0.89** | **0.94** |  |  |  |
| Slope of crown area | **0.90** | **0.92** | **0.99** |  |  |
| Slope of crown length | **0.84** | **0.95** | **0.98** | **0.97** |  |

| (c) |  |  |  |  |  |
| --- | --- | --- | --- | --- | --- |
| RLI at 1m |  |  |  |  |  |
| Total tree number | -0.50 |  |  |  |  |
| Total basal area | **-0.62** | **0.60** |  |  |  |
| Total crown area | **-0.56** | **0.82** | **0.76** |  |  |
| Total crown length | **-0.66** | **0.71** | **0.84** | **0.72** |  |

| (d) |  |  |  |  |  |
| --- | --- | --- | --- | --- | --- |
| SD of RLI at 1m |  |  |  |  |  |
| CV of tree number | **0.79** |  |  |  |  |
| CV of basal area | **0.54** | **0.59** |  |  |  |
| CV of crown area | **0.85** | **0.78** | **0.79** |  |  |
| CV of crown length | **0.84** | **0.90** | **0.63** | **0.78** |  |

Table S2. Detailed information about the range, mean, and the standard deviation of relative light intensity at 1m above the ground per plot.

| Forest age | Range of relative light intensity at 1m (%) | Mean relative light intensity at 1m (%) | Standard deviation of relative light intensity at 1m (%) |
| --- | --- | --- | --- |
| 8 | 1.12 – 8.70 | 3.11 | 1.89 |
| 9 | 0.97 – 9.51 | 5.47 | 2.25 |
| 10 | 0.82 – 15.50 | 3.09 | 3.62 |
| 15 | 1.45 – 16.72 | 7.40 | 5.13 |
| 15 | 2.47 – 10.34 | 5.27 | 2.29 |
| 21 | 0.31 – 6.49 | 2.67 | 1.86 |
| 21 | 1.23 – 2.65 | 2.66 | 0.62 |
| 21 | 1.81 – 3.94 | 1.81 | 0.46 |
| 22 | 0.51 – 1.48 | 0.94 | 0.26 |
| 23 | 0.37 – 2.51 | 1.20 | 0.62 |
| 27 | 1.24 – 6.21 | 3.23 | 1.45 |
| 30 | 1.79 – 7.85 | 4.11 | 1.64 |
| 32 | 0.45 – 6.67 | 1.14 | 0.92 |
| 32 | 0.33 – 3.89 | 2.53 | 2.01 |


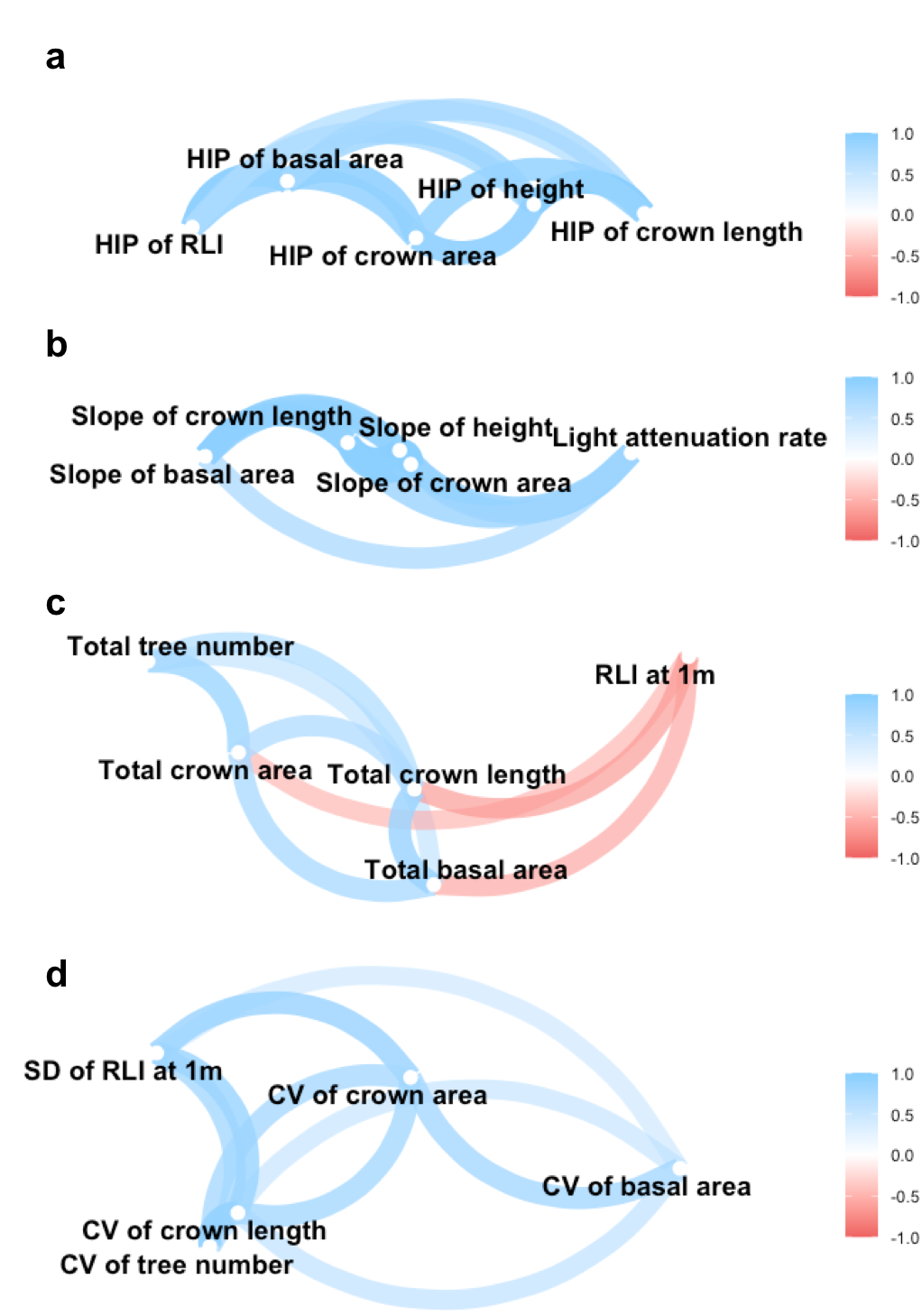


FIGURE S1. Correlation network of (a) the mean light attenuation rate (i.e., the slope of light extinction at the inflection point) and slope of cumulative forest structural attributes, (b) mean height of inflection point (HIP) of relative light intensity (RLI) across 16 sub-plots and HIP of cumulative forest structural attributes, (c) the mean RLI at 1m above the ground across 16 sub-plots and forest structural attributes per plot, and (d) standard deviation (SD) of RLI at 1m across 16 sub-plots (i.e., horizontal light heterogeneity at 1m) and coefficient of variation (CV) of forest structural attributes (i.e., horizontal heterogeneity in forest structural attributes) per plot. Darker colour intensity of the lines indicates stronger correlation. Only significant correlations are shown (p<=0.05).


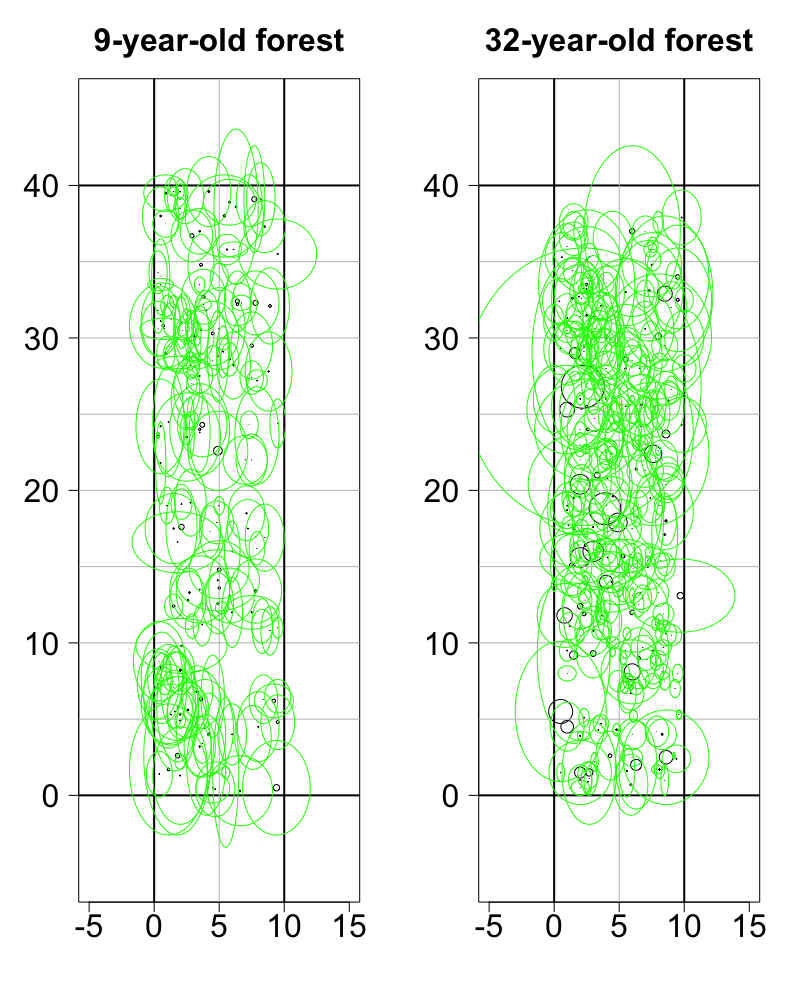


FIGURE S2. Two-dimensional representation of the forest structure of a 9-year-old (left panel) and a 32-year-old (right panel) secondary forest plots (10 m×40 m each). Crown size (green circles) and tree position (black rings) are drawn at the scale of the actual crown size and tree position. The size of black rings reflects basal area of trees.


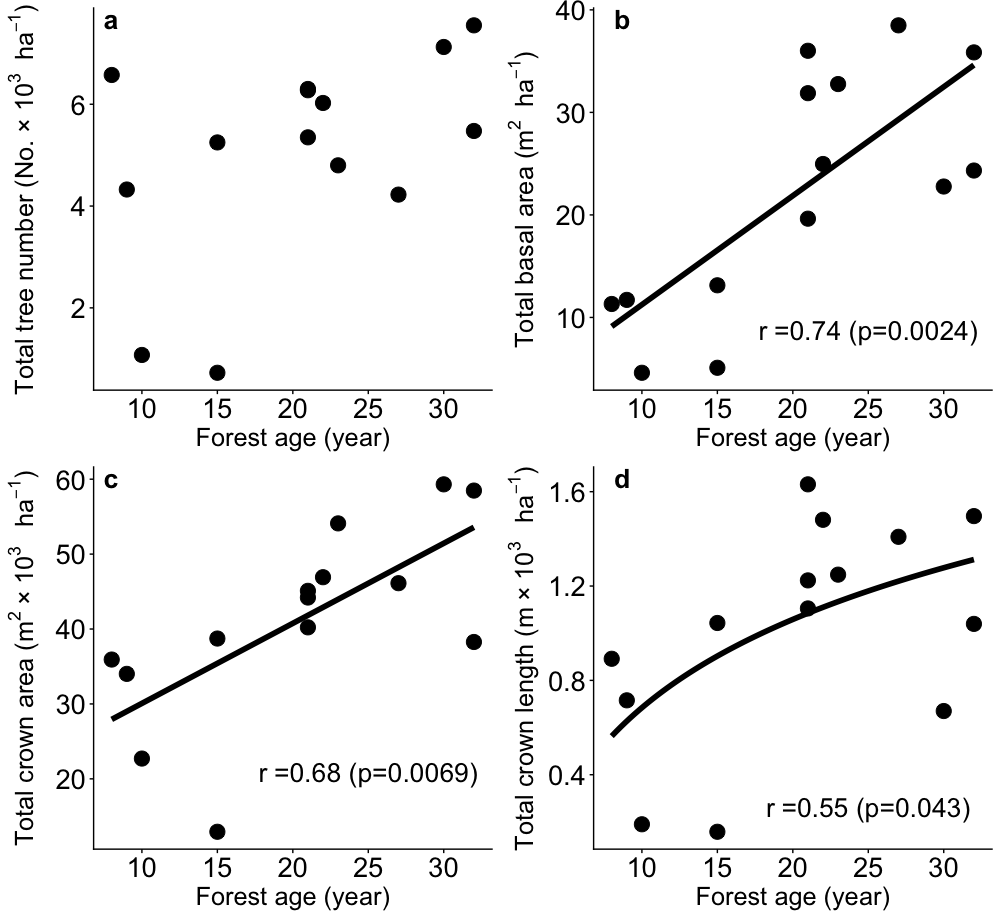


FIGURE S3. Trajectories of change of four forest structural attributes during succession. Total values of (a) tree number, (b) basal area, (c) crown area, and (d) crown length (i.e., sum of each structural attribute per plot) versus forest age. The results of a regression line (black line) and coefficient of determination (r: Pearson correlation coefficient) are shown. Regression lines are; (b) y = 1.06x + 0.62, (c) y = 1.06x + 19.39, and (d) y = 0.54log(x) - 0.56.


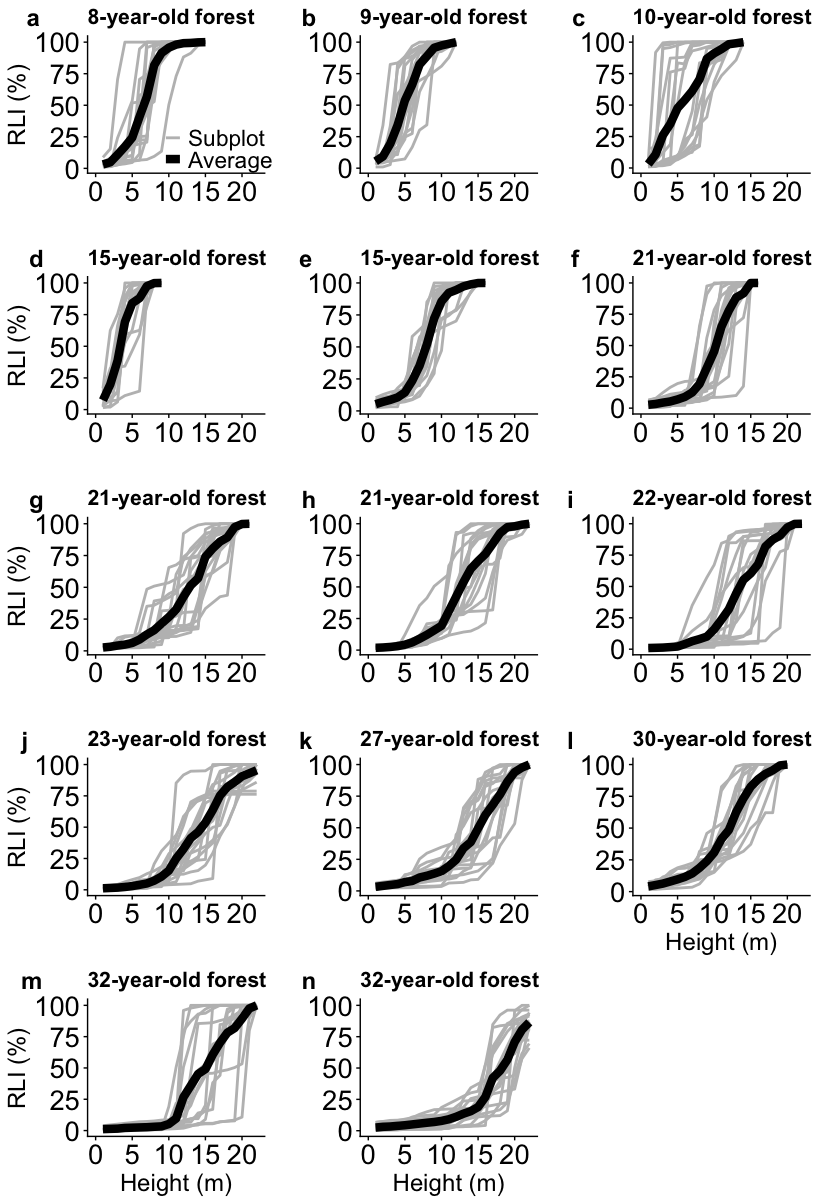


FIGURE S4. Vertical profiles of the relative light intensity (RLI) in each of the 14 studied plots. Gray lines indicate light profile for each of the 16 sub-plots, black lines indicate the average light profile across the 16 sub-plots.


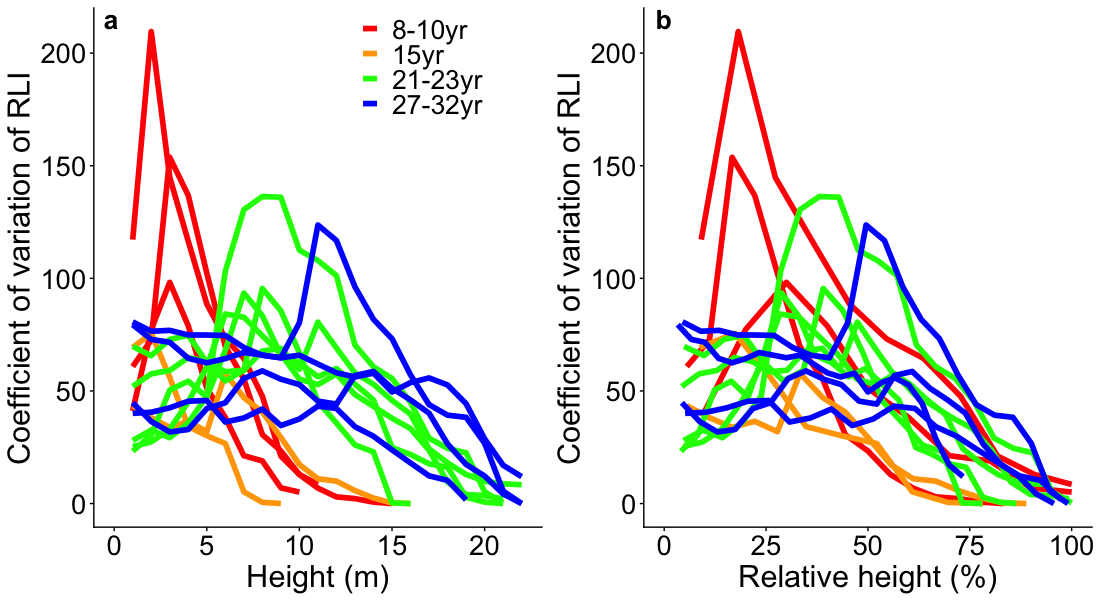


FIGURE S5. Coefficient of variation of relative light intensity (RLI, %) across 16 sub-plots at the same height versus (a) the absolute height (m) and (b) the relative height compared to the maximum canopy height per plot (%). Curves are shown for 14 plots that differ in forest age since field abandonment (as indicated by differently colours).


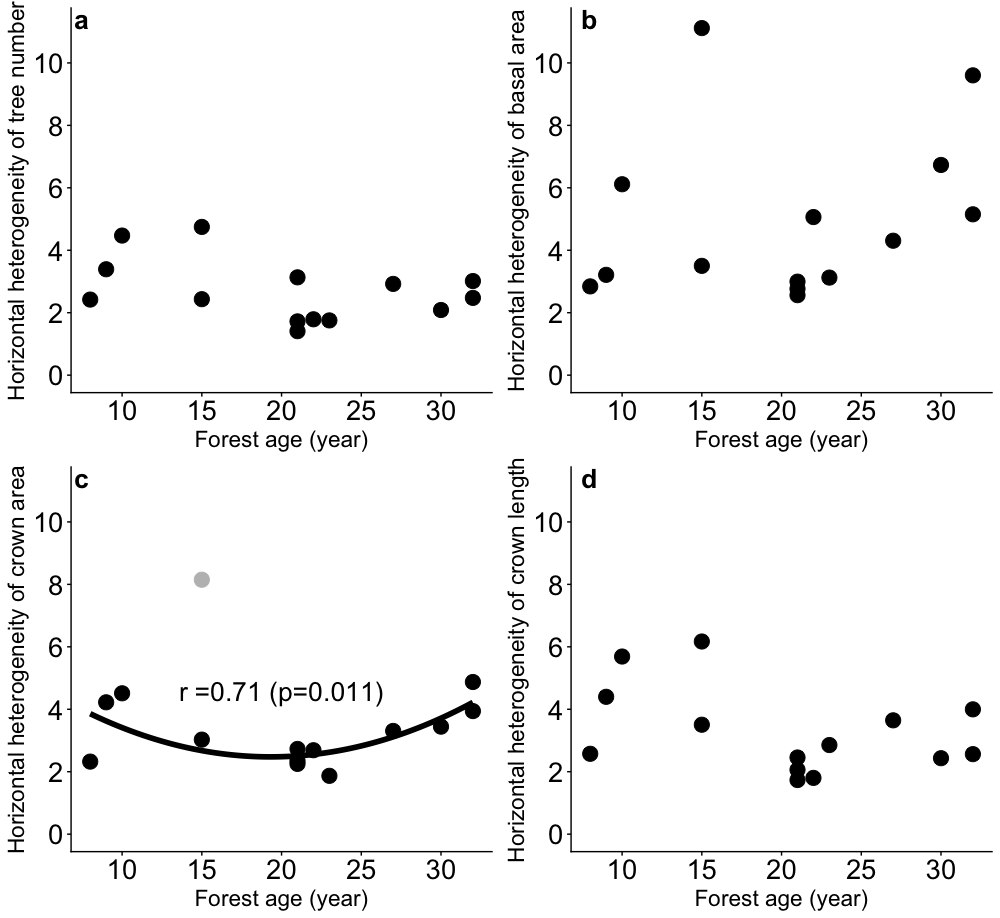


FIGURE S6. Changes of horizontal heterogeneity of forest structural attributes during succession. Horizontal heterogeneity of: (a) tree number, (b) basal area, (c) crown area, and (d) crown length (expressed as the coefficient of variation (%) in forest structural attributes across 16 sub-plots) versus forest age. The results of a fitted model (black line) and coefficient of determination (r) are shown. One plot with a grey dot in (c) is excluded for the regression as it is considered as an outlier based on Grubbs’ test (p=0.002; Grubbs, 1950). In (c), fitted model is y = 0.011x^2^ - 0.42x + 6.51.

**ADDITIONAL REFERENCE**

Grubbs, F. E. (1950). Sample Criteria for Testing Outlying Observations. *The Annals of Mathematical Statistics, 21(1)*, 27–58. 10.1214/aoms/1177729885
